# Exploring the EMG transient: the muscular activation sequences used as novel time-domain features for hand gestures classification

**DOI:** 10.3389/fnbot.2023.1264802

**Published:** 2023-11-10

**Authors:** Federico Mereu, Federico Morosato, Francesca Cordella, Loredana Zollo, Emanuele Gruppioni

**Affiliations:** ^1^Centro Protesi Inail, Vigorso di Budrio, Bologna, Italy; ^2^Unit of Advanced Robotics and Human-Centred Technologies, Università Campus Bio-Medico di Roma, Rome, Italy

**Keywords:** muscular activation sequence, onset detection, hand gesture classification, pattern recognition, transient, upper-limb amputation, hand prosthesis

## Abstract

**Introduction:**

Muscular activation sequences have been shown to be suitable time-domain features for classification of motion gestures. However, their clinical application in myoelectric prosthesis control was never investigated so far. The aim of the paper is to evaluate the robustness of these features extracted from the EMG signal in transient state, on the forearm, for classifying common hand tasks.

**Methods:**

The signal associated to four hand gestures and the rest condition were acquired from ten healthy people and two persons with trans-radial amputation. A feature extraction algorithm allowed for encoding the EMG signals into muscular activation sequences, which were used to train four commonly used classifiers, namely Linear Discriminant Analysis (LDA), Support Vector Machine (SVM), Non-linear Logistic Regression (NLR) and Artificial Neural Network (ANN). The offline performances were assessed with the entire sample of recruited people. The online performances were assessed with the amputee subjects. Moreover, a comparison of the proposed method with approaches based on the signal envelope in the transient state and in the steady state was conducted.

**Results:**

The highest performance were obtained with the NLR classifier. Using the sequences, the offline classification accuracy was higher than 93% for healthy and amputee subjects and always higher than the approach with the signal envelope in transient state. As regards the comparison with the steady state, the performances obtained with the proposed method are slightly lower (<4%), but the classification occurred at least 200 ms earlier. In the online application, the motion completion rate reached up to 85% of the total classification attempts, with a motion selection time that never exceeded 218 ms.

**Discussion:**

Muscular activation sequences are suitable alternatives to the time-domain features commonly used in classification problems belonging to the sole EMG transient state and could be potentially exploited in control strategies of myoelectric prosthesis hands.

## 1. Introduction

Nowadays, pattern recognition of surface EMG signals is extensively used in the recognition of human limb movements (gait and posture) (Yao et al., 2021; He et al., 2022) and in the classification of hand gestures (Parajuli et al., 2019; Gentile et al., 2022), for example for the control of upper limb myoelectric prostheses (Mereu et al., 2021). This control strategy consists in detecting muscle activities over a well-defined region of the arm and/or forearm and associating each pattern to predefined hand gestures using a classifier (Ortiz-Catalan et al., 2014).

Classification strategies exploit one or multiple features extracted from the EMG signal both in the time and frequency domain to control hand motion (Tkach et al., 2010; Too et al., 2017; Phinyomark et al., 2018). However, due to the high computational cost of using features extracted from the signal spectrum, time-domain features are usually preferred for real-time applications (Englehart et al., 2000; Trigili et al., 2019).

In most cases, these features are extracted after the signal reaches the steady-state (Englehart et al., 2000). This implies that a certain amount of time is required between patient intention and the classification of the gesture. As the maximum acceptable time delay between intention and classification that allows the patient not to perceive the latency of the prosthesis response is conventionally set to 300 ms (Englehart and Hudgins, 2003), it becomes essential that (i) each EMG signal reaches rapidly the steady-state and, (ii) the algorithm is transparent to the signal transition from the low to the high state to minimize classification errors in the early stage. To overcome these issues, a possible solution may be represented by adopting a pattern recognition approach based on the EMG signal acquired in the transient state. Indeed, (i) the transient EMG signal presents a deterministic pattern that could be exploited for improving classification accuracy (Yang et al., 2012; Martínez et al., 2020) and (ii) the transition from the low to the high state occurs earlier than signal stabilization (flattening) during the steady-state; therefore, the delay between patient intention and prosthesis response may be reduced.

The transient state is identified by the time instant when the signal exceeds the rest state: this is commonly defined as the onset. Onset detection algorithms differ depending on how the muscular activation thresholds are computed; such thresholds can be related to the signal magnitude at rest (Martínez et al., 2020), to a percentage of the maximum voluntary contraction (Solnik et al., 2010; Thompson et al., 2012), to the signal peak (Allison, 2003; Vaisman et al., 2010) or can be computed by mean of statistical models (Hodges and Bui, 1996; Micera et al., 1998; Xu et al., 2013, p. 1). Alternative approaches identify the onset by analyzing kinematic data acquired using instrumented gloves (Santello et al., 2002; Klein Breteler et al., 2007) or optoelectronic systems (Ricci et al., 2015).

Using the EMG signals only, the double-threshold method, proposed for the first time by Di Fabio (1987), is the most extensively used for onset detection (Hodges and Bui, 1996; Micera et al., 1998; Yang et al., 2012; Martínez et al., 2020).

According to this method, the onset is reached when the signal magnitude exceeds the magnitude in the rest state of a fixed value (typically multiple times the standard deviation of the signal in the rest state), which is maintained throughout a time window with a predefined length.

In order to use the signal onset for classification problems in applications that rely on pattern recognition, it becomes essential to identify the combination of (i) representative features of the transient state (features extraction) and (ii) a classifier, which guarantee the best classification accuracy.

Previous studies on gestures classification based on the EMG transient frequently use features and classification algorithms that are employed in steady state-based studies: most of the times the features are extracted in the time domain, like the Mean Absolute Value (MAV) (Kondo et al., 2008; Kanitz et al., 2018; Phinyomark et al., 2018; Martínez et al., 2020; D'Accolti et al., 2023); while as regards the classifiers, SVM (Yang et al., 2012; D'Accolti et al., 2023) and LDA (Phinyomark et al., 2018) are often used, with performances generally lower than the ones obtained using the signal in the steady state.

As observed in previous studies on able-body subjects, motor tasks are associated with a specific and unique pattern of muscular activation sequences. For example, Ricci et al. (2015) investigated the muscular activations of the upper limb during a combination of reaching and grasping tasks finding repeatable sequences for the recruitment of the motor control units associated with these tasks; Aeles et al. (2021) applied EMG sensors on the thigh and shank muscles to evaluate the activation patterns during trike and gait, observing that (i) a limited number of sensors is sufficient to differentiate among the phases of cycling and gait cycles and (ii) the muscular activation sequences are subject-specific. A similar approach was also used to investigate muscular activation sequences in sport-gestures such as during the swing phase in golf (Vasudevan et al., 2016) or the free throw in basketball training (Pakosz et al., 2021). Due to the previous considerations, we hypothesize that the use of muscular activation sequences for the classification of common hand gestures may improve the control of myoelectric prosthetic hands. However, to the best of the authors' knowledge, such features were never adopted in pattern recognition applications related to myoelectric prosthesis control.

Therefore, the aim of the present study was to evaluate the use of the muscular activation sequences, calculated in the EMG transient phase, as time-domain features in classification of hand gestures. Specifically, (i) a features extraction process was developed to encode the training dataset, (ii) the classification performances were computed off-line and on-line on two persons with a trans-radial amputation, (iii) a comparison with methods based on the signal envelope in the transient state (ETS) and in the steady state (ESS) was done.

## 2. Materials and methods

### 2.1. Study overview

**Figure 1:**
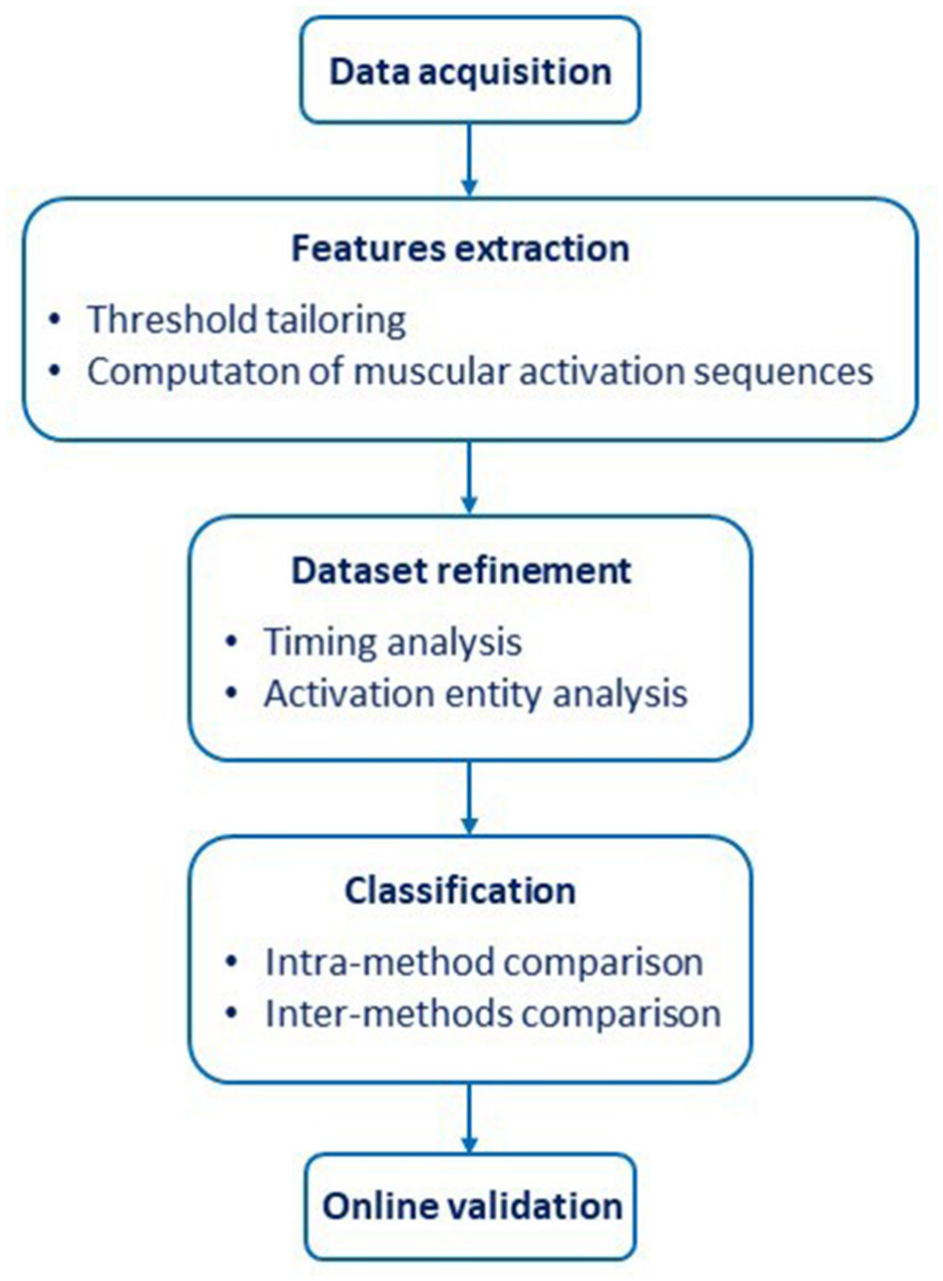
Workflow of the activities presented in the study.

The study consisted of five parts (Figure 1).

1. The EMG signals associated with four different hand gestures in healthy and amputee subjects were acquired. The hand gestures were “Spherical” (hand with all fingers closed), “Tip” (hand with thumb and index finger touching to pick up a small object), “Platform” (hand completely open and stretched), and “Point” (hand with all fingers closed, except for the index finger that is pointing); additionally, a “Rest” (relaxed hand) condition was included.
2. A feature extraction (FE) process was developed to obtain a dataset of muscular activation sequences.
3. An analysis of the activation timing and activation entity of the sequences was performed to optimize the training dataset.
4. The refined dataset was used to compare the classification accuracy of four different classifiers, namely Linear Discriminant Analysis (LDA), Support Vector Machine (SVM), Non-linear Logistic Regression (NLR) and Artificial Neural Network (ANN), commonly used in prosthetics. Then, we compared the approach based on the muscular activation sequences with approaches based ETS and ESS.
5. To validate the proposed method in a real scenario application for prosthetic control, the classification performances were evaluated with amputee subjects.

### 2.2. Experimental protocol and data acquisition

Ten able-bodied volunteers and two experts myoelectric prosthesis users with a trans-radial amputation took part in the experiments after providing the informed consent. The healthy participants (eight males and four females, aged 25÷41) were instructed to reproduce the five gestures in a natural way. The amputees both underwent a transradial amputation due to traumatic causes; P1 is a 33-year-old man, with a right amputation for 7 years and has a stump 10 cm long; P2 is a 41-year-old woman, with a left amputation for 16 years and has a stump 8 cm long. Both amputees have been using a myoelectric prosthesis for more than 6 years. Amputees were also asked to perform bimanual movements because it has been shown that it is possible to use the representation of the missing limb in the execution of gestures and that voluntary movements of a phantom arm impose behavioral constraints similar to those seen in real movement, even after the arm has been missing for more than 10 years (Franz and Ramachandran, 1998).

As there is no standard consensus about the number and the placement of the EMG sensors, a variety of experimental setups was adopted in the literature, in most cases constituted by clusters of sEMG (Castellini and van der Smagt, 2013) or HD-EMG systems (Hu et al., 2015; Stachaczyk et al., 2020) applied in different locations on the forearm. As it was demonstrated that a reduced number of sensors is sufficient to identify unique muscular patterns associated with specific motor tasks (Castellini and van der Smagt, 2013; Scano et al., 2018; Dai and Hu, 2019), we adopted a setup composed of six sEMG as it was done in previous studies (Bellingegni et al., 2017; Leone et al., 2019, 2023).

**Figure 2:**
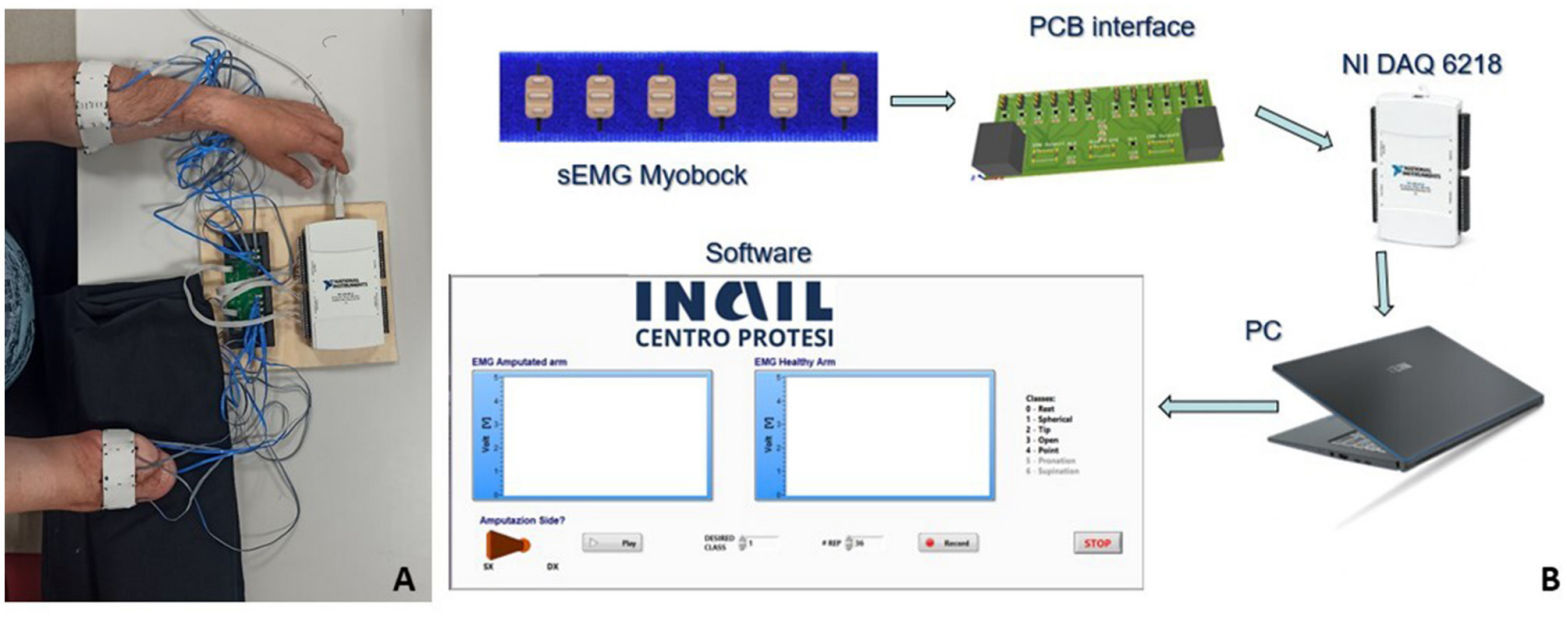
**(A)** Amputee subject during the EMG signal acquisition; **(B)** representative scheme of the experimental setup.

Therefore, the experimental setup consisted of two elastic bracelets including six equally spaced commercial sEMG sensors (Ottobock 13E200 = 50, 27 × 18 × 9.5 mm). These sensors provide the signal envelope as output. Furthermore, the sensors operate in the range of 0÷5 V with a bandwidth of 90÷450 Hz, a notch filter for the 50 Hz (European standard frequency) and a common rejection ratio higher than 100 dB. No further processing/filtering of the signal was carried out. A commercial board (NI DAQ USB 6218, National Instruments) and a dedicated LabVIEW script (v17.1, National Instrument) were used for signal acquisition. The sampling frequency was set at 1 kHz. The bracelets were placed ~5 cm below the subject's elbow before starting the test and consistently among all the participants (Riillo et al., 2014); (Figure 2). Furthermore, this position was used as it mimics what the sensors might have inside the socket of a prosthesis.

The EMG signals from both forearms were recorded simultaneously during the voluntary contractions associated with each hand gesture. Each acquisition started from a “Rest” condition, maintained for at least 500 ms, and lasted 4 s. Fifty repetitions were collected for each gesture (200 repetitions in total) and subjects were free to take short breaks between repetitions to avoid muscle fatigue. The signals acquired from the 10 able-body subjects and the sound forearm of the amputees (22 datasets in total) were used to evaluate the offline performances of the proposed method. The signals acquired from the impaired side of the amputee subjects (two datasets) were used to evaluate both the offline and the online classification accuracies.

### 2.3. Features extraction

The dataset of each subject was randomly split as follows: for each task, 80% of the dataset was used as training set, while the remaining 20% served as test set. The features were extracted from the training set following a two-steps procedure described in Sections 2.3.1 and 2.3.2, and were used to train the selected classifiers.

In the proposed method, the feature is obtained by converting the signal envelope into a discrete vector of ranking of muscular activations, named muscular activation sequence, through an optimal conversion factor obtained with the two-steps feature extraction procedure.

To test the trained classifiers, the test set was used after converting the signals envelopes into muscular activation sequences by using the optimal conversion factor computed during the training phase.

#### 2.3.1. Threshold tailoring

The first issue was to detect the thresholds associated with muscle activations:

- *TH_Low*: it represents the value beyond which muscle contraction can be considered voluntary and is unique for each dataset; this value was calculated as:

(1) $$TH_{Low}=\;\mu +x\sigma$$

where μ and σ represent the mean and the standard deviation calculated in the rest state and *x* represents a selected real value. Multiple values of *x* have been adopted in previous works where onset detection have been computed with the double threshold method. Most of them ranged between 2 and 5 (Hodges and Bui, 1996; Avila and Chang, 2014) but some authors adopted higher values (Solnik et al., 2010). As the threshold is directly related to the background noise, it is not possible to define a standard for the *x* value (Crotty et al., 2021). For this reason, to set the proper value for *x*, a tuning process was performed, investigating which value allowed to obtain a number of false activation lower than 5% on average among all the acquisitions using *x* = 2, 5, 10, 15. An activation was considered “false” if it occurred during the first 100 ms of acquisition, i.e., during the rest phase. In our dataset the signal amplitude in the rest phase was on average 0.012 V (min value = 0.006 V, max value = 0.021 V) with a standard deviation of 0.0007 V (min value = 0.0002 V, max value = 0.0017 V). Therefore, to obtain a number of false activations lower than 5% on average among all the acquisitions, we used *x* = 15. Indeed, using *x* = 2, 5, 10, we obtained a number of false activations higher than 50, 12, and 7%, respectively. To avoid considering spurious activations, the lower threshold was considered reached only if the signal exceeded TH_Low throughout a time window of 50 ms (hereafter SoA—Start of Activation). In case the SoA was not detected, the muscle underlying that specific sensor was considered “Not Active” (NA). This procedure allowed identifying both the SoA and which EMG signal never exceeded TH_Low within each acquisition.

**Figure 3:**
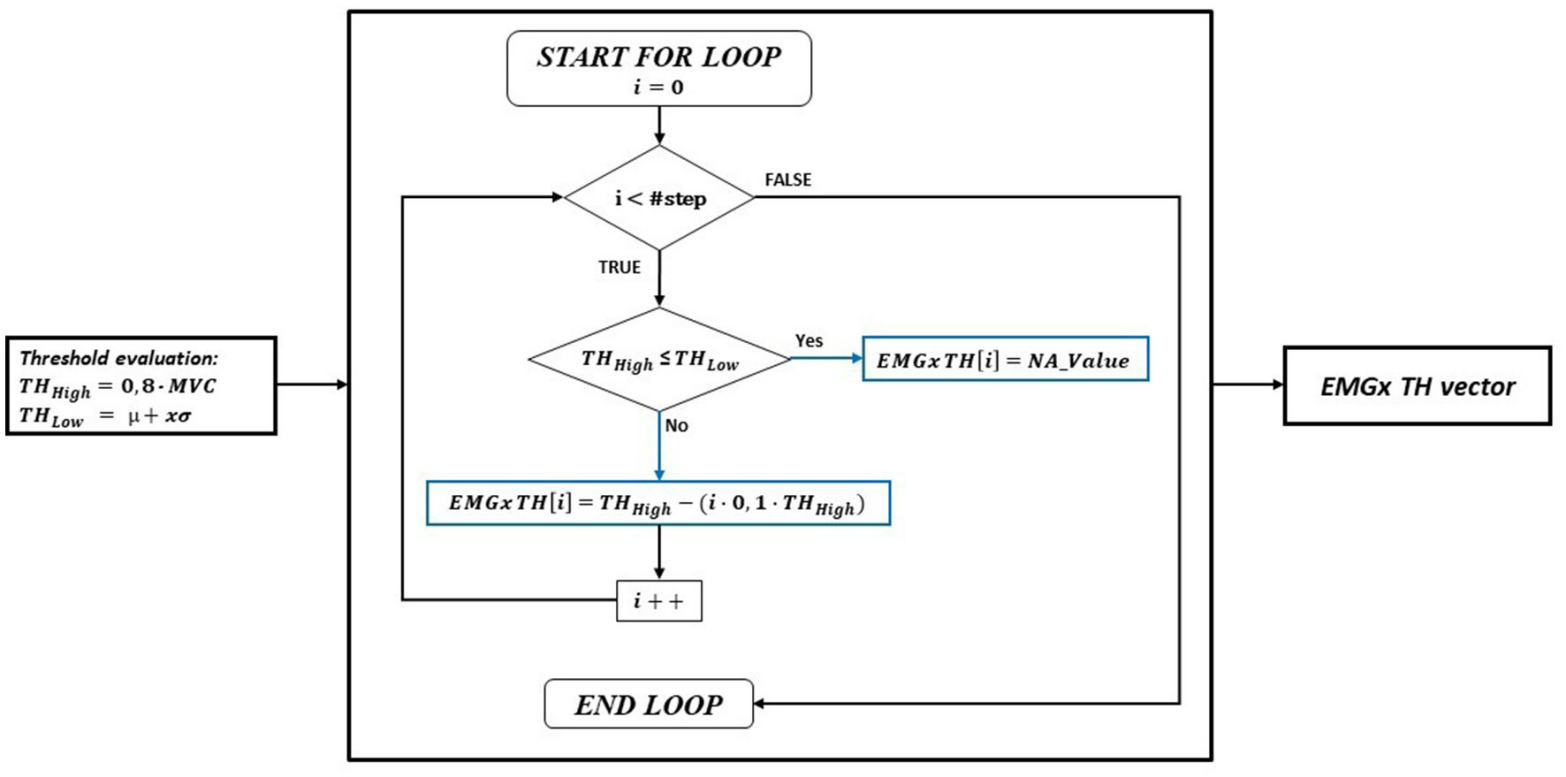
Flow chart of the TH_High evaluation process. The process is shown for a generic EMGx.

- *TH_High*: it represents the value above which the muscle could be considered actually active (hereinafter, any reference to “activation” refers to the exceed of the TH_High). If an EMG was considered NA from the previous step, the relative TH_High value was set to “NA_Value” (practically corresponding to a value not reachable by the EMG signal, i.e., higher than the full-scale value) for that specific acquisition. To compute the TH_High for each EMG sensor and for each task (resulting in a 4 × 6 matrix) an iterative process was developed. The iterative process consisted in a nested loop. The process was applied for each task separately. The 80% of the EMG signal peak, extracted within a time frame of 300 ms from the SoA, was computed for each sensor and averaged over the acquisitions, resulting in a 1 × 6 maximum TH_High vector representing the starting point of the iterative process. The outer loop variable was the #step, indicating how many times the maximum TH_High must be decreased inside the inner loop for each EMG separately, in consecutively iterations. To identify an active muscle within 300 ms from the SoA, each threshold value was sequentially decreased by 10% at each iteration, as long as the signal was higher than 50% of the peak. Consequently, the maximum #steps was 5. For example, using #step = 3 the TH_High possible values, for each EMG, were 80, 72, and 64% of the maximum EMG signal. Consequently, using #step = 3, the number of threshold combinations computed in the inner loop was 3^6^ (#step^#EMG^). Basically, all the possible combinations of TH_High thresholds were computed for each #step. The creation of the TH_High vector for each EMG can be represented as shown in Figure 3.

The initial value of TH_High was the 80% of the MVC. If TH_High had a lower value than TH_Low, NA_Value was used to fill the EMGx TH_High vector. Otherwise, the initial value of TH_High was the first value of the EMGx TH_High vector (i.e., for *i* = 0). Then, for a number of iterations equal to #step, this value was decreased inside the for loop by 10% on each iteration. At every cycle, the EMGx TH_High vector was filled with the new TH_High value or with NA_Value, depending on whether the new TH_High value is lower than the TH_Low or not.

#### 2.3.2. Computation of the muscular activation sequences

**Figure 4:**
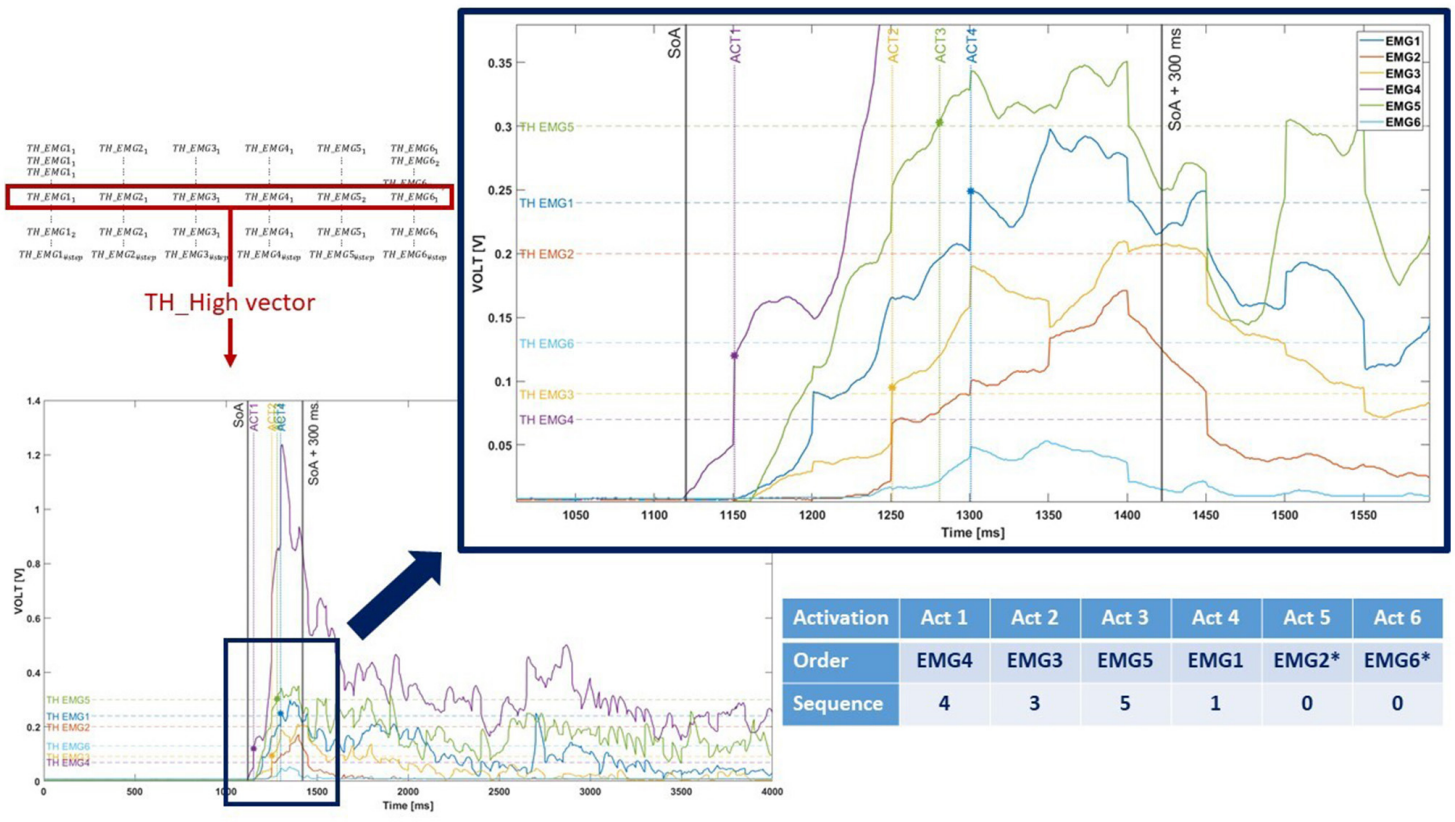
Graphical representation of the muscular activation sequence encoding. As shown in the picture, two EMG signals (EMG2 and EMG6, marked with ^*^) never exceed the TH_High. Therefore, they are represented with “0” value in the sequence.

A muscular activation sequence was obtained by evaluating the time instant when each EMG signal exceeded the correspondent TH_High value. The #EMG sensor was used to encode the sequence: for example, if the “EMG4” signal was the first to exceed its TH_High, the first element of the sequence vector was set to “4”. If an EMG did not exceed its TH_High or if the TH_High value was previously set to NA_Value, the relative element of the vector was set to “0” and appended at the end of the sequence (Figure 4).

This procedure was performed for each acquisition and each TH_High, generating a total of #step^6^ possible muscular activation sequences. To obtain a single representative TH_High vector (1 × 6), the one that allowed obtaining the most repeated muscular activation sequence was selected. This process was performed for each task resulting in a (4 × 6) matrix of representative TH_High and consequently in a (4 × 6) matrix of activation sequences for each acquisition and each #step.

### 2.4. Dataset refinement

As the duration of the transient state of the EMG signal from the low state to the high state may vary considerably between acquisitions (even for the same task) (Reaz et al., 2006), only muscular activations within 300 ms from the SoA were considered eligible for the classification. Moreover, as muscular activation is strictly related to the hand gesture [having that not all muscle fibers are recruited during the voluntary contraction associated with a specific task (Clamann, 1981)], the activation timing (i.e., how many activations occurred within 300 ms from the SoA) and the activation entity (i.e., how many EMG were active after the iterative process) were evaluated for all the muscular activation sequences associated with the representative TH_High.

The result of this process allowed for refining the dataset before training, generating a dataset of partial (i.e., truncated) activation sequences. Further details can be found in Section 3.1.

### 2.5. Classification

#### 2.5.1. Intra-method comparison

Four different typically used classifiers were selected, namely NLR, SVM (linear kernel), ANN and LDA (Kotsiantis et al., 2007; Bellingegni et al., 2017) classifiers. All the computations were made with Matlab (v. 2021b, MathWorks). To evaluate which #step provided the better classification accuracy and the relative classification timing, each classifier was trained and validated using different input datasets (i.e., one for each #step).

The classifiers' parameters matched those from earlier publications by the authors (Bellingegni et al., 2017; Leone et al., 2019, 2023).

A linear and binary supervised classification approach called logistic regression uses the logistic function to estimate the likelihood that a class will belong to it. To achieve a NLR the creation of additional input, namely interaction terms, is needed. Similar in previous studies, extra polynomial features were included, which were derived as a combination product of the initial input features. Then, by comparing the distribution P(y|x) with a decision threshold (equal to 50%), class labels can be predicted.

The supervised ANN in question is a Multi-Layer Perceptron (MLP), where each node, or neuron, in the design implements a logistic function. An input layer, one or more (up to five) hidden layers with the same number of neurons, and an output layer with one neuron for each class that needs to be classified make up the network design.

A Radial Basis Function (RBF) kernel is used in the SVM.

A one *vs. all* approach was implemented to address with the multi-class classification problem because LDA is a binary classification algorithm.

#### 2.5.2. Inter-methods comparison

In order to compare the proposed method with methods based on the EMG signal envelope in the transient (ETS) and stationary state (ESS), the initial EMG signals were elaborated prior to be used as input training dataset. In particular:

- For ETS the input dataset was represented by the signal envelope comprised in a time window of 300 ms starting from SoA (similarly to Kuiken et al., 2009; Kanitz et al., 2018; Zhang et al., 2019).
- For ESS the input dataset was represented by the MAV extracted over a moving window of 100 ms in the flatten portion of the signal whose starting point was approximated as the first absolute signal peak among the 6 EMG signal envelopes within a single acquisition.

A comparison between the classification performances of the three methods was performed thereafter with a non-parametric (Mann-Whitney *U*-test) or parametric (paired *t*-test) test, depending on the result of a normality test.

In order to compare the proposed method and the ESS method in terms of classification timing, the elapsed time between the SoA and the class selection timing of both methods was assessed as follows:

- For the proposed method, the elapsed time was measured as the temporal distance between the SoA and the end of the partial activation sequence (i.e., the last activation);
- For ESS, an underestimation of the elapsed time was computed as the temporal distance between the SoA and the beginning of the steady state approximated as the instant when the first absolute signal peak was reached among the 6 EMG signal envelopes within a single acquisition.

This evaluation was performed for each #step.

### 2.6. Online validation

To evaluate the performance of the classifier in an online application, an additional test was conducted with the impaired subjects, focusing on the amputee side. The test consisted of the real-time classification of the selected tasks, randomly presented to the subject via a dedicated software and acquired five times each. The motion completion rate (MCR) and the motion selection time (MST) were used as indicators of the online performances, as done in Kuiken et al. (2009). MCR is the percentage of successfully completed motions out of the total attempted motions. The MST is the time taken to correctly select a target motion and was defined as the time from movement onset to the first correct classification (Kuiken et al., 2009).

## 3. Results

The series of 200 acquisitions (i.e., 50 repetitions for each task) were correctly acquired from each subject without any missing value.

### 3.1. Dataset refinement

The timing analysis showed that muscular activation occurred within 300 ms for three EMG signals per activation sequence, on average among the four tasks, with #step = 4, 5. The first activation occurred within 150 ms from the SoA on average among the tasks and the #steps (Table 1).

**Table 1:** The elapsed time within the first three activations and the SoA.

	**First act (ms)**	**Second act (ms)**	**Third act (ms)**
#step = 2	142.53	246.96	333.22
#step = 3	128.81	228.81	316.87
#step = 4	115.91	216.11	289.46
#step = 5	104.61	207.25	282.97

From the analysis of the activation entity, it resulted that muscular activation occurred for three EMG signals per activation sequence, on average among the four tasks: “Point” was the task with the least number of activations on average (i.e., 2, approximation to the closer integer), while “Close” was the task with the highest number of activations on average (i.e., 5, approximation to the closer integer). Hence, it was decided to calculate the partial activation sequences using the threshold vectors obtained for each of the tasks, since it is required to compute the activation sequences using the best threshold vector related to each gesture once the SoA has been identified. By using this method, it was possible to identify the activation order that was acquired with each of the four threshold vectors, resulting in a matrix of four activation sequences (4 × 6) that are all typical of the same task. Therefore, the dimension of the matrix of activation sequences was refined to include the first three elements of the muscular activations sequence vector for each task, resulting in a (4 × 3) matrix of partial activation sequences. Eventually, to encode each acquisition with a single row vector, the rows of the matrix of partial activation sequences were sequentially appended, resulting in a (1 × 12) vector for each acquisition. The resulting matrix was used as input dataset for the classification.

### 3.2. Classification

#### 3.2.1. Intra-method comparison

The classification accuracy of the selected classifiers (NLR, SVM, ANN, LDA), calculated for each #step, is shown in Table 2. Overall, the highest performance was obtained with the NLR classifier. The highest accuracy was obtained when 4 steps were adopted. Indeed, with #step = 4 the differences between the performance of the NLR classifier and the other classifiers were statistically significant (*p* < 0.0001).

**Table 2:** The classification accuracy of the selected classifier obtained using the partial activation sequences (columns 1–4) and the EMG signal in the transient as training dataset.

	**#step = 2**	**#step = 3**	**#step = 4**	**#step = 5**	**ETS**	**ESS**
NLR	90.6 ± 5.3	91.0 ± 4.7	93.1 ± 3.5	91.3 ± 3.8	92.2 ± 3.3	98.4 ± 0.9
SVM	84.9 ± 6.9	86.4 ± 6.0	87.2 ± 5.4	88.2 ± 5.1	90.0 ± 2.7	97.9 ± 1.1
ANN	86.3 ± 7.5	87.3 ± 4.7	86.9 ± 5.7	86.8 ± 5.3	87.6 ± 4.0	97.8 ± 1.2
LDA	84.3 ± 9.3	85.6 ± 7.1	87.2 ± 6.9	85.8 ± 5.7	78.7 ± 4.5	93.1 ± 2.7

All the values are expressed in percentage (%).

#### 3.2.2. Inter-methods comparison

The performance of the same classifiers trained with ETS were comparable with the performance obtained with the proposed method. In particular, the accuracy differences were lower than 1% using NLR and ANN and lower than 3% with SVM; however, using the LDA, performances obtained with the proposed method were significantly higher (almost 9% higher, *p* = 0.0004, Mann-Whitney *U*-test) (Table 2).

The accuracy obtained using the ESS were generally higher than the proposed method (with #step = 4) regardless of the classifier: in particular, such a difference was higher than 5% using NLR and LDA, and higher than 10% using SVM and ANN. Looking at the classification timing, the class selection with ESS occurred 496.03 ± 270.53 ms after the SoA, and up to 213 ms later than the proposed method on average among the tasks (Table 3).

**Table 3:** The time difference between the classification obtained with the proposed method and the one based on the steady state.

**#step = 2**	**#step = 3**	**#step = 4**	**#step = 5**	**ESS**
195 ± 97	201 ± 95	213 ± 87	211 ± 91	Reference

All values are expressed in milliseconds (ms).

#### 3.2.3. Online validation

Overall, the offline performances of amputee subjects were higher than the performances of the able-body subjects for each classifier and each #step. The largest accuracy was obtained with the NLR classifier and #step = 4 on average (Table 4). In particular, the differences between the accuracies obtained using activation sequences (with #step = 4) and ETS were lower than 2.5% using NLR, lower than 20% with SVM and ANN and about 25% using LDA. As for able-bodied, the highest accuracies were found with ESS.

**Table 4:** The classification accuracy of the selected classifier obtained using the partial activation sequences and the EMG signal envelope in the transient as training dataset for each trans-radial amputee.

**P1**	**#step = 2**	**#step = 3**	**#step = 4**	**#step = 5**	**ETS**	**ESS**
NLR	91.84	92.07	95.83	97.82	92.07	99.11
SVM	90.02	90.45	90.55	95.03	72.17	96.90
ANN	90.33	92.36	93.06	94.36	71.83	96.86
LDA	92.04	93.11	93.60	95.31	64.62	94.13
**P2**	**#step** = **2**	**#step** = **3**	**#step** = **4**	**#step** = **5**	**ETS**	**ESS**
NLR	92.47	95.74	95.79	90.66	94.57	97.06
SVM	93.03	91.39	90.89	92.36	70.28	94.71
ANN	90.14	91.36	90.23	89.29	72.06	94.93
LDA	92.20	91.08	90.58	90.36	69.32	92.82

All the values are expressed in percentage (%).

Looking at the classification timing, the class selection was reached earlier using the activation sequences, in comparison with ESS: in particular, for P1, classification occurred 627 ms (#step = 2), 655 ms (#step = 3), 725 ms (#step = 4) and 735 ms (#step = 5) earlier on average among the tasks; for P2, classification occurred 379 ms (#step = 2), 440 ms (#step = 3), 500 ms (#step = 4) and 491 ms (#step = 5) earlier on average among the tasks.

**Figure 5:**
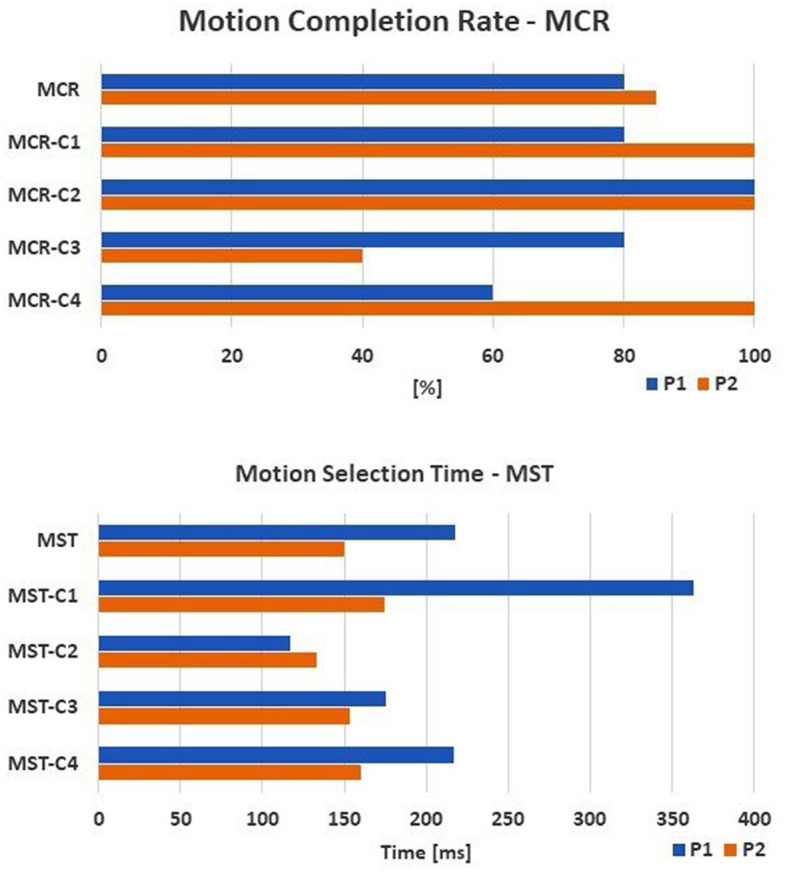
The online performance of the amputee subjects are shown: the motion completion rate—MCR (up) and the motion selection time—MST (down) computed for each task. The tasks are: C1, spherical; C2, tip; C3, platform; C4, point.

Concerning the online performances, an MCR of 80 and 85% was reached by P1 and P2, respectively. The “Tip” class was recognized in 100% of cases. The MST was 218 ± 129 ms and 150 ± 59 ms for P1 and P2 respectively (Figure 5).

## 4. Discussion

The aim of the present study was to evaluate the robustness of the muscular activation sequences for the classification of hand gestures based on pattern recognition and to assess the performances of four common classifiers trained with the proposed features. This represents a novelty in this field, as muscular activation sequences were never investigated as features for myoelectric prosthesis control so far.

The EMG signals were collected from both the forearms of 10 able-bodied subjects and 2 amputees. An algorithm for the identification of the muscular activation sequences associated with four selected tasks starting from the EMG signal in the transient state was developed. The algorithm consisted of three main parts: (i) a double threshold mechanism was used for the identification of the onset; (ii) the identification of a representative activation sequence for each gesture, and (iii) the encoding and refinement of the single acquisitions.

The refined training dataset was used as input for four common classifiers, namely NLR, SVM, ANN and LDA, and the classification performances were compared. Furthermore, these results were compared with the classification performances obtained using the signal envelopes in the transient state and in the steady state.

Eventually, the proposed algorithm was tested on two persons with trans-radial amputation in an online application.

A reduced setup consisting of only six sEMG sensors was adopted. It has been demonstrated in past studies that the same setup was sufficient to classify a limited number on classes with a good accuracy (Scano et al., 2018). Therefore, in perspective, such a setup could be easily adjusted for applications in a real scenario of myoelectric prosthesis control, which, so far, rely on a limited number of sensors.

The classification accuracy obtained with the activation sequences were comparable to the ones obtained using ESS (NLR, step = 4) and in line with past studies that relied on ETS (offline accuracy > 90%) (Solnik et al., 2010; Yang et al., 2012; Ricci et al., 2015; Kanitz et al., 2018; Martínez et al., 2020; D'Accolti et al., 2023; Leone et al., 2023), demonstrating to be suitable or even better alternatives to the commonly used features.

Considering able-bodied, the largest difference in terms of accuracy was observed between NLR (step = 4) and LDA (step = 4), i.e., almost 9%. This may be a reflection of the ability of the activation sequences to be less sensitive to the highly non-linear nature of the EMG signal in the transient state. This behavior was further confirmed with the amputees, in which differences between accuracy were lower than 2% on average using the NLR classifier, and up to 25% using LDA.

Overall, offline performances of healthy and amputee subjects achieved by using the activation sequences were comparable (Tables 2, 4). The reasons may be dual. First, from a physiological standpoint, the muscular activation sequences mirror the motor fiber recruitment strategy people adopt during any kind of motion, which are extremely subject-dependent (Clamann, 1981). After amputation, the resected muscles are rearranged in the stump and re-innervation occur, generating new neurological pathways and consequently new motor control strategies for the end-effector, i.e., the prosthesis hand (Wheaton, 2017; Gunduz et al., 2020). In our case, the amputee subjects were experienced users (at least 6 years of myoelectric use), with a (theoretically) consolidated fiber recruitment strategy. Second, the activation sequences allow shifting the feature extraction issues from the EMG amplitude in the transient state, which may vary considerably within the same subject and motion task depending, among others, on the muscle volume (Wheaton, 2017) to an order of muscular activations, which may be more repeatable among recruitment strategies of muscular fibers. Therefore, despite the extremely limited amputee sample prevent any inferential consideration, it may be possible that the activation sequences represent a reliable classification feature for prosthesis control purposes. Unfortunately, it has been never investigated previously for the classification of hand gestures as it was done for evaluating motion patterns in tasks that involve upper limbs (Micera et al., 1998; Xu et al., 2013; Ricci et al., 2015) and lower limbs (Aeles et al., 2021) or for studying sport exercises (Vasudevan et al., 2016; Pakosz et al., 2021). Although the good preliminary results, further investigations are mandatory to assess if the robustness of the proposed features is stable over time or is affected by the “classical” limb orientation problem (Campbell et al., 2020) or by the increase of the number of classes.

In the study, we quantified the time delay between class selection with the proposed method and ESS, which was underestimated as the first EMG signal peak among the six EMG signals used for motion detection. The classification delay was up to 213 ms for the able-bodied, and up to 735 and 500 ms for the amputees (P1 and P2, respectively), on average among tasks. Such a result arises the possibility to implement a variety of control strategies, which combine multiple classifiers in cascade or exploit the signal transient for classification and the steady state for non-classification purposes. For example, past studies used features of the transient state to classify grasping task and features of the steady state for carrying out a finer control of the end-effector speed or position (Kanitz et al., 2018; Martínez et al., 2020; D'Accolti et al., 2023). Concerning the classification times, in Kanitz et al. (2018) and D'Accolti et al. (2023) the transient-based classifier provides the output after 300 and 200 ms, respectively, starting from the onset, in line with the proposed method, i.e., 289 ms (with #step = 4).

The online performances were evaluated with two common indicators, i.e., MST and MCR. The obtained MCR was comparable with the results in D'Accolti et al. (2023) (between 75.6 and 89.2%) and higher than the ones obtained in Kanitz et al. (2018), i.e., 95% on average. It must be noted that both the studies differed considerably from the present work in terms of the number and type of electrodes and the feature extraction methods. Moreover, in Kanitz et al. (2018) the accuracy evaluation was performed after an optimization process to reduce the limb position effect on the hand gesture classification, which was not investigated in the present work. Therefore, a direct comparison with our findings is troublesome.

To the Author's best knowledge both the MST and the MCR were evaluated in a single study related to hand gesture classification involving two trans-humeral amputees. Five hand gestures were classified. The MST was 220 ± 60 ms, and MCR of 86.9 ± 13.9% (Kuiken et al., 2009), therefore comparable with our findings.

The study presents some limitations. Due to the nature of the EMG transient, the activation bursts are unique for each hand motion and acquisition. Therefore, in order to generate the initial dataset, each hand gesture must be acquired several times (50 times per task in the present work). Compared with the pattern recognition algorithms based on ESS, which usually require a reduced number of acquisitions, our method necessitate of an extensive training. Further investigations could be done to optimize the number of acquisitions. As previously mentioned, the limb orientation effect was not assessed. Further analysis should be conducted to evaluate how this factor affect the fiber recruitment strategies and, consequently, the generated muscular activation sequences. Eventually, the amputee sample should be extended to evaluate the reliability of the proposed method, including subjects with a different experience in myoelectric prosthesis use.

## 5. Conclusion

To summarize, we presented a novel feature extraction method based on the muscular activation sequences extracted in the transient state of the EMG signal during voluntary muscle contraction.

The method was tested on 4 different commonly-used classifiers using signals acquired from 10 able-body subjects (offline test) and two subjects with a unilateral trans-radial amputation (offline and online test).

We demonstrated that muscular activation sequences are suitable alternatives to the time-domain features commonly used in classification problems belonging to the sole EMG transient state. Moreover, looking at the ESS or mixed (EST and ESS) methods, the sequences have the potential to anticipate the gesture selection by totally excluding the signal steady state in the classification process. In this regards, we quantify the time-delay between class selection with our method and a method based on the ESS.

Due to the reduced number of EMG sensors and the good performances obtained both in offline and online applications, our pattern recognition approach could be further investigated in order to be implemented in the control of myoelectric prostheses.

## Data availability statement

The raw data supporting the conclusions of this article will be made available by the authors, without undue reservation.

## Ethics statement

The studies involving humans were approved by CE-AVEC Sant'Orsola Malpighi, Bologna, Italy; Protocol Code: CE18138—CP-PPRAS1/1-01. The studies were conducted in accordance with the local legislation and institutional requirements. The participants provided their written informed consent to participate in this study.

## Author contributions

FMe: Conceptualization, Data curation, Investigation, Methodology, Writing—original draft. FMo: Conceptualization, Data curation, Methodology, Writing—original draft. FC: Supervision, Writing—original draft. LZ: Supervision, Writing—review & editing. EG: Conceptualization, Supervision, Writing—review & editing.
